# Heavy metals in cannabis: plant contamination and effects on cannabinoid production

**DOI:** 10.1186/s42238-026-00460-6

**Published:** 2026-07-27

**Authors:** Itamar Beigel, Sivan Shiponi, David Meiri, Punya Nachappa, Nirit Bernstein

**Affiliations:** 1 Institute of Soil, Water and Environmental Sciences, Agricultural Research Organization- Volcani Institute, Rishon LeZion, Israel; 2 The Robert H. Smith Faculty of Agriculture, The Hebrew University of Jerusalem, Rehovot, Israel; 3 Laboratory of Cancer Biology and Natural Drug Discovery, Faculty of Biology, Technion-Israel Institute of Technology, Haifa, Israel; 4 Department of Agricultural Biology, Colorado State University, Fort Collins, CO 80523 USA

**Keywords:** Cannabis, Heavy metals, Ni, Cd, Co, Pb, Secondary-metabolites, Contamination

## Abstract

**Background:**

Exposure of cannabis to heavy metals may interfere with metabolic pathways of cannabinoid biosynthesis and compromise its medical quality. Furthermore, inflorescence contamination with heavy metals presents a critical challenge for the production of safe pharmaceutical-grade cannabis, since it poses a significant health risk to consumers. The present study therefore evaluated the hypotheses that heavy-metal exposure affects cannabinoid production, leads to inflorescence-contamination, and compromises the cannabis plant function; and that the responses are genotype-dependent and heavy-metal dose-dependent.

**Methods:**

To evaluate the hypotheses, we studied responses of four ‘drug-type’ medical cannabis cultivars to a cocktail of four heavy-metals (Cd, Pb, Ni, Co), in three concentrations each (0, 1, 5µM), and analyzed translocation and accumulation patterns of the heavy-metals in the plant organs, and the resulting impact on cannabinoid production and the plant’s physiological integrity.

**Results:**

The results confirmed effects of the heavy metals on cannabinoid production, with a heavy-metal concentration threshold, and genotypic variability, thus confirming the hypotheses.

**Conclusions:**

The roots accumulated the highest levels of heavy metals, demonstrating an avoidance strategy of exclusion from sensitive shoot organs; and the root-to-shoot translocation factor was Ni > Cd, Co > Pb demonstrating heavy-metal specificity. The accumulation patterns revealed that plant exposure to moderate-low heavy metal concentrations (5µM) poses health concerns, as the inflorescences’ Cd and Ni concentrations were above the WHO-permitted threshold for medical plant consumption.

## Introduction

The bioactive secondary metabolites in cannabis are produced and accumulate in glandular trichomes, reaching the highest concentrations in female inflorescences, which are the plant organs used for consumption (Bernstein et al. 2019). A major concern regarding the safety of cannabis plant products is the potential for their contamination during cultivation and post-harvest processing by various health compromising compounds, including heavy metals (Montoya et al. 2020; Amendola et al. 2021). When consumed, heavy metals tend to bioaccumulate in the body organs and pose a serious health hazards (Soni et al. 2024).

Heavy metals in the environment are derived from various sources including natural, industrial, and agricultural (Yalin et al. 2023). Although they are known to adversely affect the environment and living organisms, several heavy metals including Fe, Mn, Zn, Cu are micronutrients, which are essential for plant growth and development. However, exposure to high concentrations of heavy metals can cause agricultural yield losses by imposing negative effects on plant function and morpho-physiology, including altered membrane functions, modification of stomatal function, photosynthesis, respiration, metabolism, and enzyme activity of (Gill 2014; Ashfaque et al. 2016). Plants have adapted mechanisms to cope with heavy metal stress, which involve exclusion of metals in isolated compartments, reduced uptake, storage, detoxification, chelation, *in-planta* trafficking, defense mechanisms against oxidative stress, and synthesis of stress-related proteins (Baig et al. 2020). Of potential relevance to cannabis, and to the safety of the cannabis plant products, a common heavy-metal detoxification method in tolerant plants is their compartmentation in trichomes (Li et al. 2023). Heavy metals were found in drug-type cannabis products (Dryburgh et al. 2018; Dubrow et al. 2021), and smoke (Moir et al. 2008; Granata et al. 2024), but no information is available on the accumulation and transport of heavy metals in drug-type medical cannabis, and their effect on secondary metabolism.

Heavy metals can contaminate cannabis during cultivation and post-harvest. During cultivation, the plant can accumulate heavy metals through acquisition from the soil, tainted fertilizers, irrigation water, and foliage sprays of fertilizers or pesticides containing trace levels of heavy metals. Post-harvest, heavy metals can contaminate the product during inflorescences processing from metallic equipment, and during extraction, depending on the method and equipment used.

It is known for several plant species that trichomes may accumulate heavy metals as a heavy metal detoxification strategy (Karabourniotis et al. 2020; Koul et al. 2021). In cannabis, the highest density of trichomes- the secondary metabolite producing organs- is found in the plant’s inflorescences, which are the plant’s medical yield, highlighting the importance of increasing our understanding of heavy-metal translocation and accumulation in the plants, for increasing the safety of the cannabis plant product.

In addition to their direct toxic effects on consumers, heavy metals that accumulate in the plant may also affect the medical quality of the cannabis inflorescence product, by affecting the production of cannabinoids, the cannabis-specific biologically active compounds. Heavy metals are known to affect the production of secondary metabolites in plants. For example, exposure to Cd and Cu increased phenolics, flavonoids and saponin contents in *Gynura procumbens* (Ibrahim et al. 2017); exposure to Pb increased total phenols and flavonoids contents in *Prosopis farcta* (Zafari et al. 2016); and in tomato seedlings, exposure to Ni increased total phenolics, flavonoids, and anthocyanins (Jahan et al. 2020). Only limited information is available concerning effects of heavy-metal exposure on cannabinoid production, and the information available is only for Hemp- cannabis. The few available studies mainly focused on exposure to Cd. In Hemp, high Cd levels (2.5–25 mg/L) decreased or had no effect on cannabinoid production, depending on the cannabis cultivar and Cd concentration (Marabesi et al. 2023a, b). Exposure to a mix of Cd, Cr, and Ni had no effect on cannabinoid production (Citterio et al. 2003), Yin et al. (2022) reported that a CBD response gene is associated with Cd exposure in hemp. Beyond that, information on the effects of heavy metals on cannabinoid production is scarce, and this issue should be further studied to help guide cultivation practices for the benefit of consumers.

Cannabinoid production in medical cannabis is sensitive to mineral nutrients (Saloner and Bernstein 2022a), demonstrating a susceptibility to inorganic mineral elements. Supply of nitrogen (Saloner and Bernstein 2021), phosphorous (Shiponi and Bernstein 2021a) potassium (Saloner and Bernstein 2022b), magnesium (Morad and Bernstein 2025), and the timing of mineral supply (Saloner et al. 2024), alter the secondary-metabolite profile in cannabis, and induce metabolic shifts (Song et al. 2023).

Closing the knowledge gap regarding heavy-metal homeostasis, and the effects of heavy metals on secondary metabolite production in drug-type cannabis is therefore of importance to ensure a safe and standardized medical product for the health of the consumer. The goals of this study were therefore to evaluate potential effects of heavy metals on function and cannabinoid production in medicinal (drug-type) cannabis plants, and to characterize uptake and distribution patterns of heavy metals in the plant organs. The hypotheses guiding the work plan were: (i) The cannabinoid profile in the inflorescences is affected by root exposure to heavy metals, and the extent of the effect is dose dependent. (ii) The accumulation of heavy metals in the cannabis plants differs between plant organs and is affected by the concentrations supplied to the plants. (iii) Heavy metals affect the plant morpho-physiology, and the effect is dose- dependent. (iv) There is a genetic variability in the response of the cannabis plants to heavy metals. To test these hypotheses, we studied physiological and developmental responses of the plants to a cocktail of four heavy metals (Cd, Pb, Ni, Co), each in three concentrations (0, 1, 5 µM), and analyzed the accumulation patterns of the heavy metals in the plant organs, and cannabinoid concentrations. The obtained results are required for the development of cultivation practices for the production of a safe medicinal products.

## Materials and methods

### Plant material and growing conditions

Four medicinal cannabis cultivars QJ, Dov, LD, PO-10 (PO) (Canndoc LTD, Herzelia, Israel) were used as model plants for the study. The plants were generated by vegetative propagation, from cuttings from a single mother plant for each cultivar. The cuttings were rooted in coconut fiber plugs (Jiffy international AS, Kristiansand, Norway) for 14 days, and after an additional week of hardening, 15 plants were selected for uniformity for each cultivar. The selected uniform plants were transplanted into 3 L plastic pots, one plant per pot, with perlite (2-1-2, Agrekal, Habonim, Israel) as the growing medium. The plants were grown at the vegetative stage for 3 weeks under a long photoperiod (18:6 h light/dark), with Metal Halide bulbs (Solis Tek Inc, Carson, California; 25.9 mol m d^− 1^) as the light source, at a light intensity of 200–340 µmol m^− 2^ s^− 1^. Temperature and relative humidity in the cultivation room were 25 °C and 45–60%, respectively. The short photoperiod (12/12 hr. light/dark) was applied 5 days after the initiation of the heavy metal treatments (17 days after the beginning of the vegetative growth). Light was supplied using High Pressure Sodium bulbs (Greenlab by Hydrogarden, Petah Tikva, Israel) with a light intensity of 750–1050 µmol m^−2^s^− 1^.

Irrigation was supplied using 1 L h^− 1^ discharge- regulated drippers (Netafim, Tel-Aviv, Israel), set to generate 35% leachate in each irrigation event. Fertilizers were supplied with the irrigation solution at each irrigation. The concentration of nutrients in the fertigation solution were (in mg L): 160 N (14 N-NH_4_^+^, 146 N-NO_3_^−^ ), 175 K, and 30 P, which are the optimal concentrations we identified in previous studies for cannabis (Saloner and Bernstein 2021, 2022b; Shiponi and Bernstein 2021a), with 110 Ca^2+^, 35 Mg^2+^, 85 S-SO_4_^−2^, 1.7 Fe^2+^, 0.8 Mn^2+^, 0.10 B, 0.4 Zn^2+^, 0.04 Cu^2+^, and 0.03 Mo^2+^. pH was measured using a pH meter (Cyberscan pH 1500, Eutech Instruments Europe B.V., Nijkerk, Netherlands) and EC with a conductivity meter Cyberscan CON 1500 (Eutech Instruments Europe B.V., Nijkerk, Netherlands). The pH of the nutrient solution was adjusted to 6.0-6.1, and EC was 1.8 mS cm^− 1^. The experiment was terminated at the maturation stage accepted for chemical maturity in the studied cultivars, when about 20% of the glandular trichomes head were amber. This occurred after 56 days under short photoperiod for the cultivars Dov and QJ, and 63 days for PO and LD.

### Experimental treatments

One week before the termination of the vegetative period (following 12 days under the long photoperiod), plants from each cultivar were randomly divided to 3 treatment groups of 5 plants each. Each group received one of three concentrations of a heavy-metal mixture containing Cd, Co, Ni, and Pb, which was added to the fertilization solution. Each of the four heavy metals was added to the fertigation solution in equal concentrations of 0 (non-treated control), 1 µM, or 5 µM. The heavy metal treatments were initiated 5 days before the switch to the short-day (flowering-inducing) photoperiod and were maintained throughout the reproductive stage.

### Plant development and biomass

Plant height, number of nodes on the main stem, and stem diameter were measured at the end of the reproductive stage as described by Saloner and Bernstein ( 2021). The data were collected from 5 replicate plants per treatment. At the termination of the experiment, 60 and 67 days after the start of the heavy metal treatments for QJ + Dov and PO + LD, respectively, the plants were sampled destructively. Each plant was divided into inflorescences, inflorescence-leaves, leaves, stem, and roots. The samples were weighted, washed twice in deionized water (roots were washed 3 times), blotted dry, and dried in 64˚C for 48–144 h until no further weight change occurred, and then weighed again to determine dry weight. The inflorescence leaves were hand-trimmed prior to drying.

### Heavy metal analysis

For the analysis of the concentration of the studied heavy metals (Cd, Co, Ni and Pb) in the plant organs, the dried samples of each organ from each plant were ground to a homogeneous powder, and 0.1–0.105 g was taken for elemental analysis. The samples were acid digested with HClO_4_ (70%), and HNO_3_ (65%) as described by Bernstein et al. (2005, 2019), and the analysis was preformed using a dual-view High-Resolution ICP-OES spectrometer PlasmaQuant PQ9000 (Analytic Jena, Germany).

### Translocation factor

For the evaluation of the effect of the heavy metals (Co, Ni, Cd, Pb) supply to the plant, on root-to-shoot translocation of the heavy metals in the plant, a translocation factor was calculated using Eq. 1, following Shiponi and Bernstein (2021b):

**Eq. 1** $$\begin{aligned}&\:Translocation\:Factor\:\left(TF\right)\\&=\frac{Concentration\:of\:the\:heavy\:metal\:in\:the\:shoot}{Concentration\:of\:the\:heavy\:metal\:in\:the\:root}\end{aligned}$$  

### Physiological parameters

The plants were sampled for physiological analysis on day 37 after the initiation of the heavy metal treatments (49 days after transplanting). All measurements were conducted on the youngest fully mature leaves on the main stem. Following the experimental design, 5 independent replicas were taken per treatment each from a different replicated plant, and the reported results are averages ± SE. For photosynthetic pigment analysis, the selected leaf was excised from the plant, washed twice in distilled water and gently blotted dry. Five-discs, 0.6 mm in diameter, were removed from the leaf, placed in 0.8 ml ethanol (80%) and stored in -20 ˚C until analysis Ignat et al. (2013). Pigment extraction from the tissue, and the analyses of pigment concentrations in the extracts, were conducted as is described by Bernstein et al. (2010a). For Membrane leakage analysis, following dissection from the plant and washing in distilled water, the middle leaflet was immediately placed in a 50 ml test-tube, filled with 30 ml distilled water. The tubes were shaken for 24 h and the EC of the solution containing the leaf was measured using a conductivity meter (Cyberscan CON 1500, Eutech Instruments Europe B.V., Nijkerk, Netherlands). The tubes were then autoclaved for 30 min, cooled down to room temperature and shaken again for 1 h, and EC of the solutions was measured again. Membrane leakage was calculated as described by Shoresh et al. (2011). Gas exchange parameters (photosynthesis rate, stomatal conductance, transpiration rate, intercellular CO_2_ concentration, and intrinsic water use efficiency (WUEi) were measured on day 37 after the initiation of the heavy metal treatments (at the 5th week of flowering), using a Licor 6400 XT system (LI-COR, Lincoln, NE, USA) (CO_2_ concentration: 400 mg L-1, PPFD: 500 µmol (m2s)-1) (Saloner and Bernstein 2023). The measurements were conducted in the morning, 1 h after the first irrigation and the initiation of the light period.

### Cannabinoid analysis

At harvest, i.e., day 56 after the initiation of the reproductive stage for the cultivars Dov and QJ, and day 63 for PO and LD, the top inflorescence of the main stem was sampled for cannabinoid analyses. The inflorescences were hand-trimmed and air-dried under 19.5 °C and 45% relative humidity for two weeks, following conventional commercial practices. For the analyses, each sample was ground to a powder with a manual plastic grinder, 50 mg of the ground tissue was placed in a 50 ml tube, 10 ml EtOH ABS AR (Gadot-Group, Netanya, Israel) was added to each tube, and the tubes were shaken in a reciprocal shaker for 1 h. The generated extract was filtered with a 0.22 μm filter (PVDF, Bar-Naor Ltd, Ramar Gan, Israel).

Cannabinoid analysis was performed using an UltiMate 3000 ultra-high-performance liquid chromatography coupled with an ultraviolet-visible diode array detector (UHPLC/UV) system (Thermo Scientific, Bremen, Germany) according to the method described by Berman et al. (2018). In short, chromatographic separation was performed using a HALO C18 Fused-Core column (2.7 μm, 150 × 2.1 mm), with a HALO guard column (2.7 μm, 5 × 2.1 mm), and a ternary A/B/C multistep gradient (solvent A: water with 0.1% acetic acid, solvent B: acetonitrile with 0.1% acetic acid, and solvent C: methanol). Cannabinoid quantification was performed using external calibration with respective analytical standards, with ten-point calibration curves (R^2^ > 0.99) in the range of approximately 0.02–500 µg/ml. Data acquisition was performed at 263 nm and 200–400 nm photodiode array (Lipson Feder et al. 2021).

### Statistical analysis

The experiments followed a random experimental design, with five independent replicates per treatment. The data were analyzed using one-way analysis of variance (ANOVA), followed by Tukey’s HSD post-hoc test for separation of means. The data met the assumptions of normality and homogeneity of variances. The analysis was performed with the Jmp software (Jmp package, version 9, SAS 2015, Cary, NC, United States).

## Results

### Plant growth and development

**Fig. 1:**
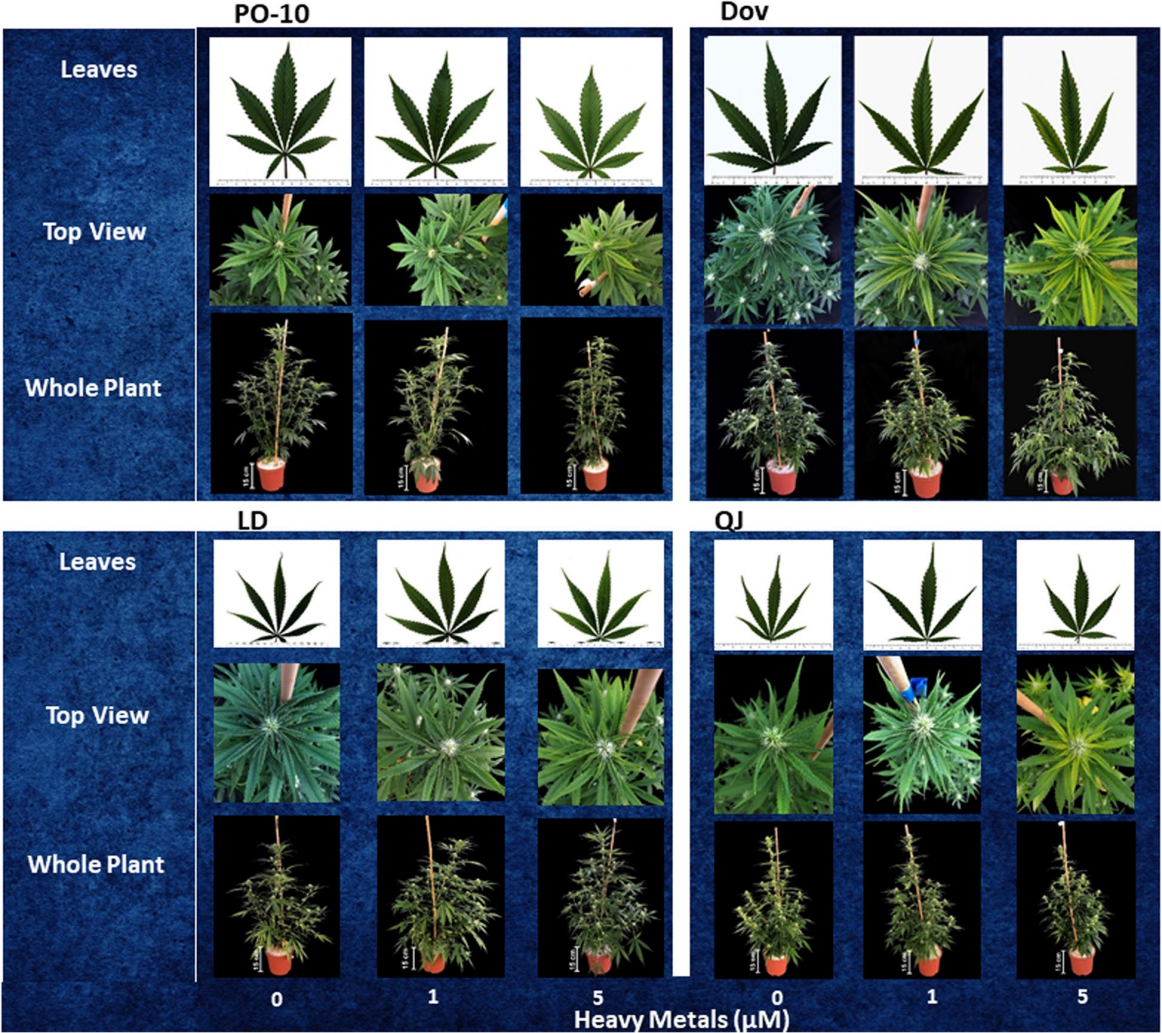
Effect of the heavy metals on plant visual appearance in four medical cannabis cultivars: QJ, PO, LD, Dov. The plants were exposed to a mix offour heavy metals (Pb, Co, Cd, Ni) at three concentrations: 0, 1, 5 μM. Leaves (top raw), top view of the plants (second raw), and whole plant (bottom raw).Leaves images are of the youngest-fully developed leaf on the main stem. All images were taken 27 days after the initiation of the heavy metal treatments

The visual appearance of leaves, top view, and whole plants at the 4^th^ week of the reproductive stage, reflects the plant response to the level of heavy metals supplied (Fig. 1). Exposure to the heavy metals cocktail induced visual damage symptoms of chlorosis in the leaves and inflorescence leaves in all four cultivars, suggesting a direct toxicity response to the heavy metals, or an induced deficiency or toxicity of plant nutrients. The visual symptoms were clearly apparent in the plants exposed to the higher heavy metal concentration (5 µM), whereas only slight changes compared to the control were apparent under the lower heavy metal concentration (1 µM).

### Morphological characteristics and biomass accumulation

**Fig. 2:**
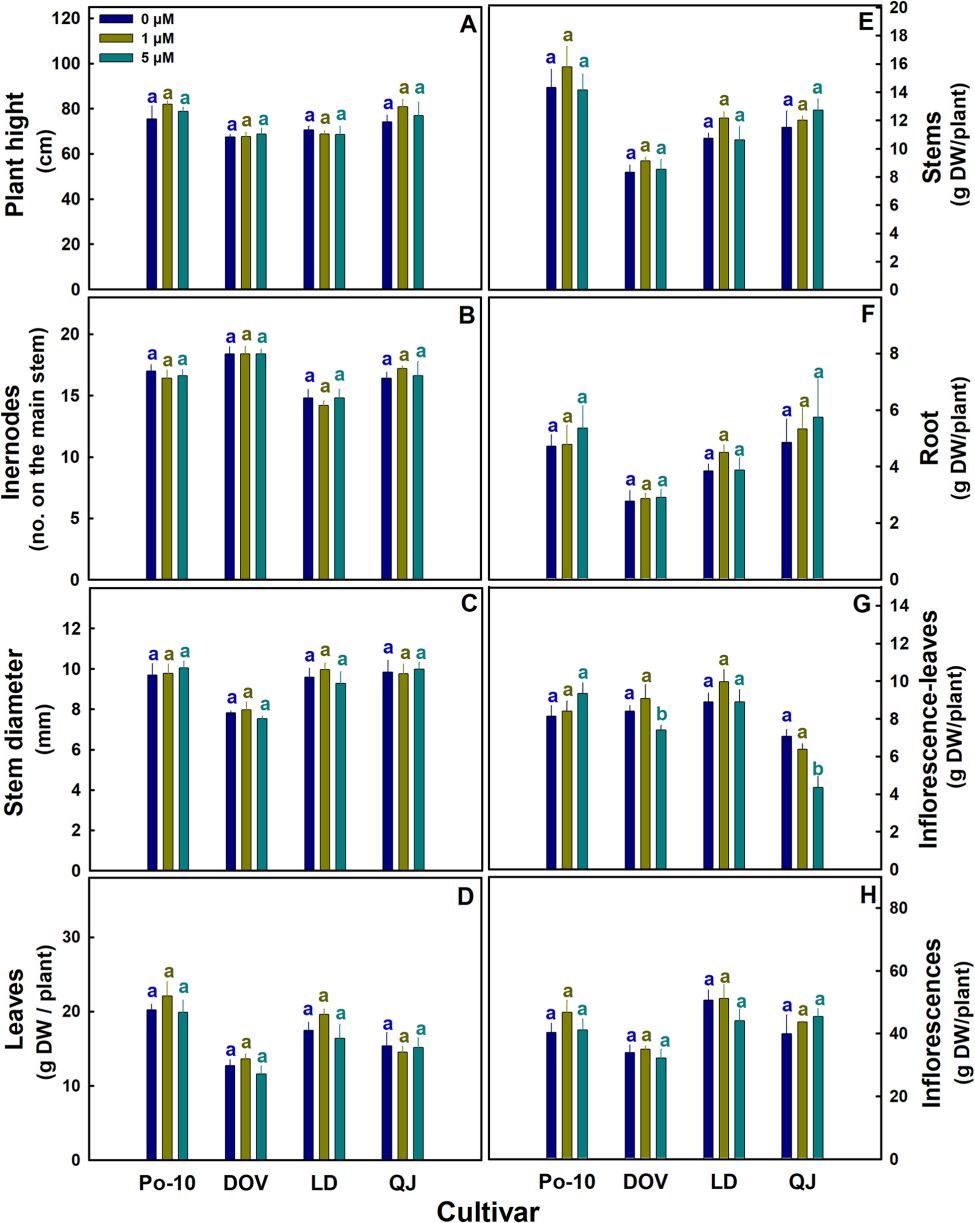
Effect of the heavy metals on plant morphological characteristics and plant biomass in four medical cannabis cultivars: QJ, PO, LD, Dov. The plants were exposed to a mix of four heavy metals (Pb, Co, Cd, Ni) at three concentrations: 0, 1, 5 µM. Plant height (**A**), No. of internodes on the main stem (**B**), stem diameter (**C**), Dry biomass of leaves (**D**), stems (**E**), root (**F**), inflorescences leaves (**G**), and inflorescences (**H**). The presented results are average ± SE (*n* = 5). Different small letters above the means signify significant differences between treatments within a cultivar, by Tukey HSD test at α = 0.05

In all four cultivars, the heavy metal treatments had no effect on plant development. Plant height, the number of internodes on the main stem and stem diameter were not significantly different between treatments in any of the examined cultivars (Fig. 2A-C). Similarly, no effects were identified on biomass accumulation of individual plant organs (Fig. 2D-F, H), except for the inflorescence leaves in the cultivars Dov and QJ, which were significantly smaller in the high-metal treatment compared to the other two treatments (Fig. 2G).

### Gas exchange and physiological characteristics

**Fig. 3:**
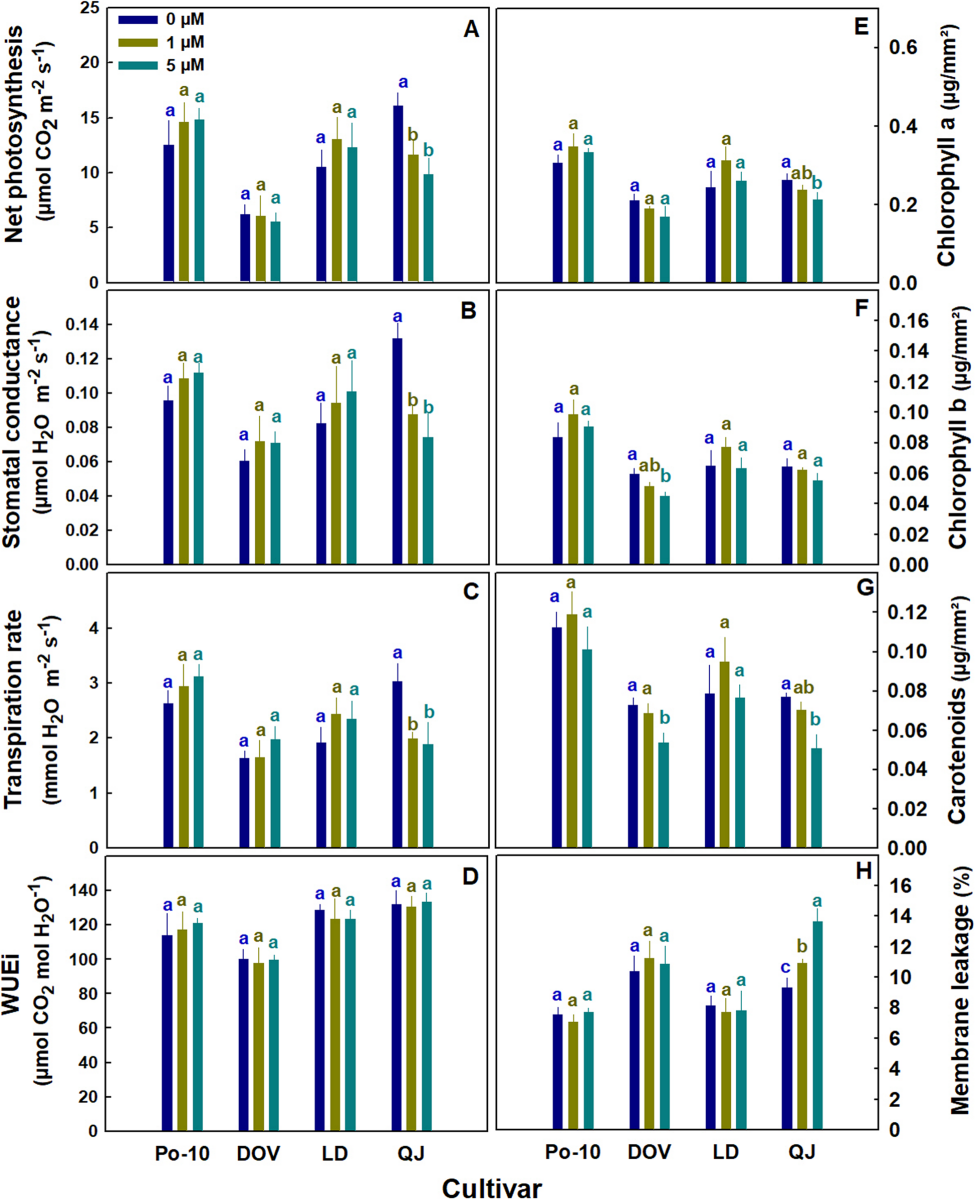
Effect of the heavy metals on gas exchange parameters (**A**-**C**), water use efficiency (WUEi) (**D**), concentration of photosynthetic pigments (**E**-**G**), and membrane leakage (**H**), of four medical cannabis cultivars: QJ, PO, LD, Dov. The plants were exposed to a mix of four heavy metals (Pb, Co, Cd, Ni) at three concentrations (0, 1, 5 μM). Presented results are average ±SE (n=5). Different letters above the bars signify significant differences between treatments within a cultivar by Tukey HSD test at α=0.05

Gas exchange parameters were affected only in the cultivar QJ (Fig. 3). Exposure to heavy metals reduced net photosynthesis, stomatal conductance and transpiration rate in QJ (Fig. 3A, B, E). Photosynthetic pigments were affected as well by the heavy metal treatments in Dov and QJ (Fig. 3E-G). In QJ, chlorophyll a and carotenoid concentrations were reduced by the 5 μm treatment, and so were the chlorophyll b and carotenoid concentrations in Dov.

Membrane leakage is an indicator of the oxidative damage that affects membrane integrity (Bernstein et al. 2010b). The cultivar QJ demonstrated an increase in membrane permeability with the heavy metal treatments; it increased already by the low heavy metal treatment (1µM) (Fig. 3H). Membrane integrity was not affected in the remaining cultivars.

### Cannabinoid profiles

**Fig. 4:**
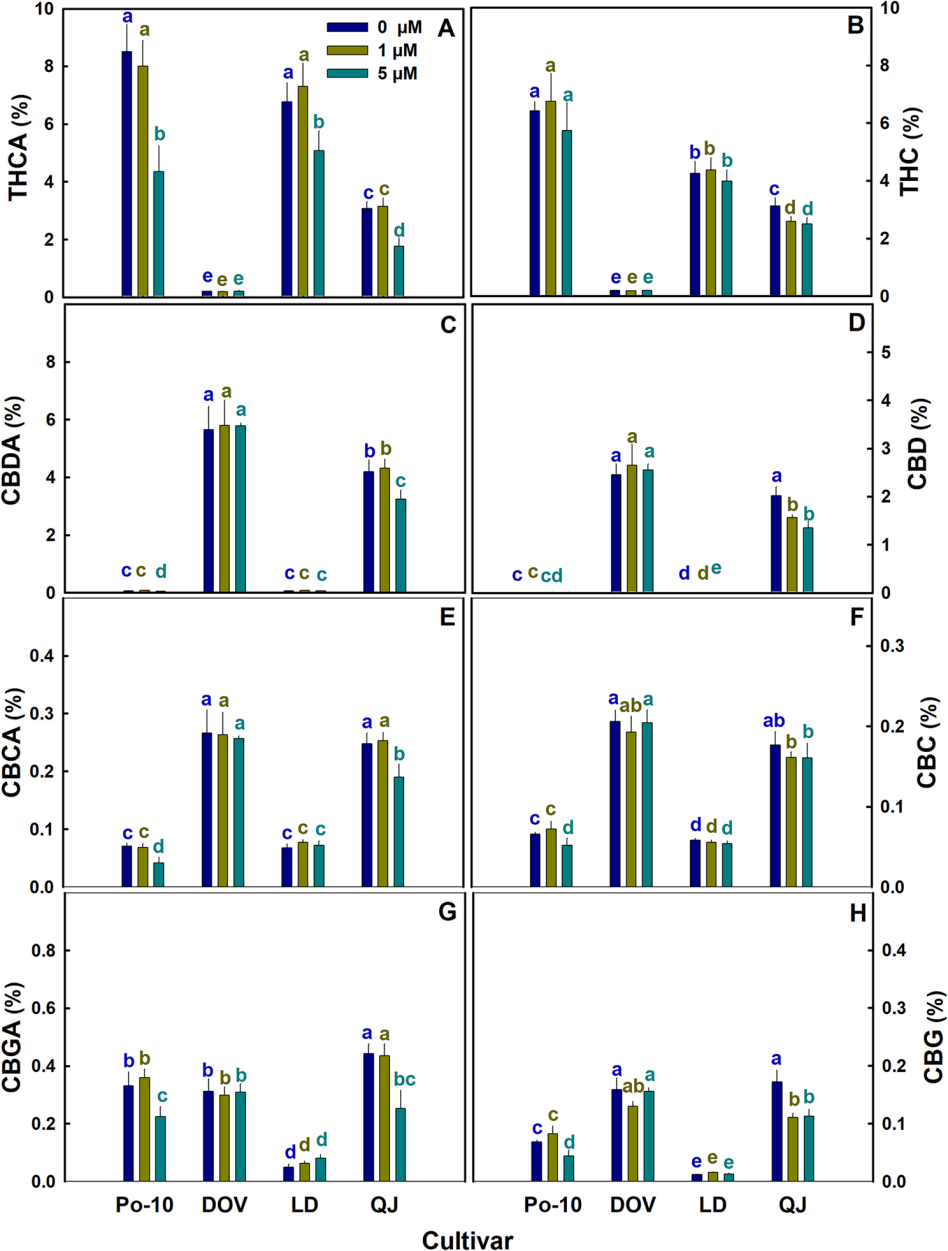
Effect of the heavy metals on cannabinoid concentrations in inflorescences of four medical cannabis cultivars: QJ, PO, LD, Dov. The plants were exposed to a mix of four heavy metals (Pb, Co, Cd, Ni) at three concentrations (0,1, 5 µM). Tetrahydrocannabinolic acid (THCA) (**A**), tetrahydrocannabinol (THC) (**B**), cannabidiolic acid (CBDA) (**C**), cannabidiol (CBD) (**D**), cannabichromenic acid (CBCA) (**E**), cannabichromene (CBC) (**F**), cannabigerolic acid (CBGA) (**G**), and cannabigerol (CBG) (**H**). The presented results are average ± SE (*n* = 5), % W/W. Different letters above the means signify significant differences between means by Tukey HSD test at α = 0.05

**Fig. 5:**
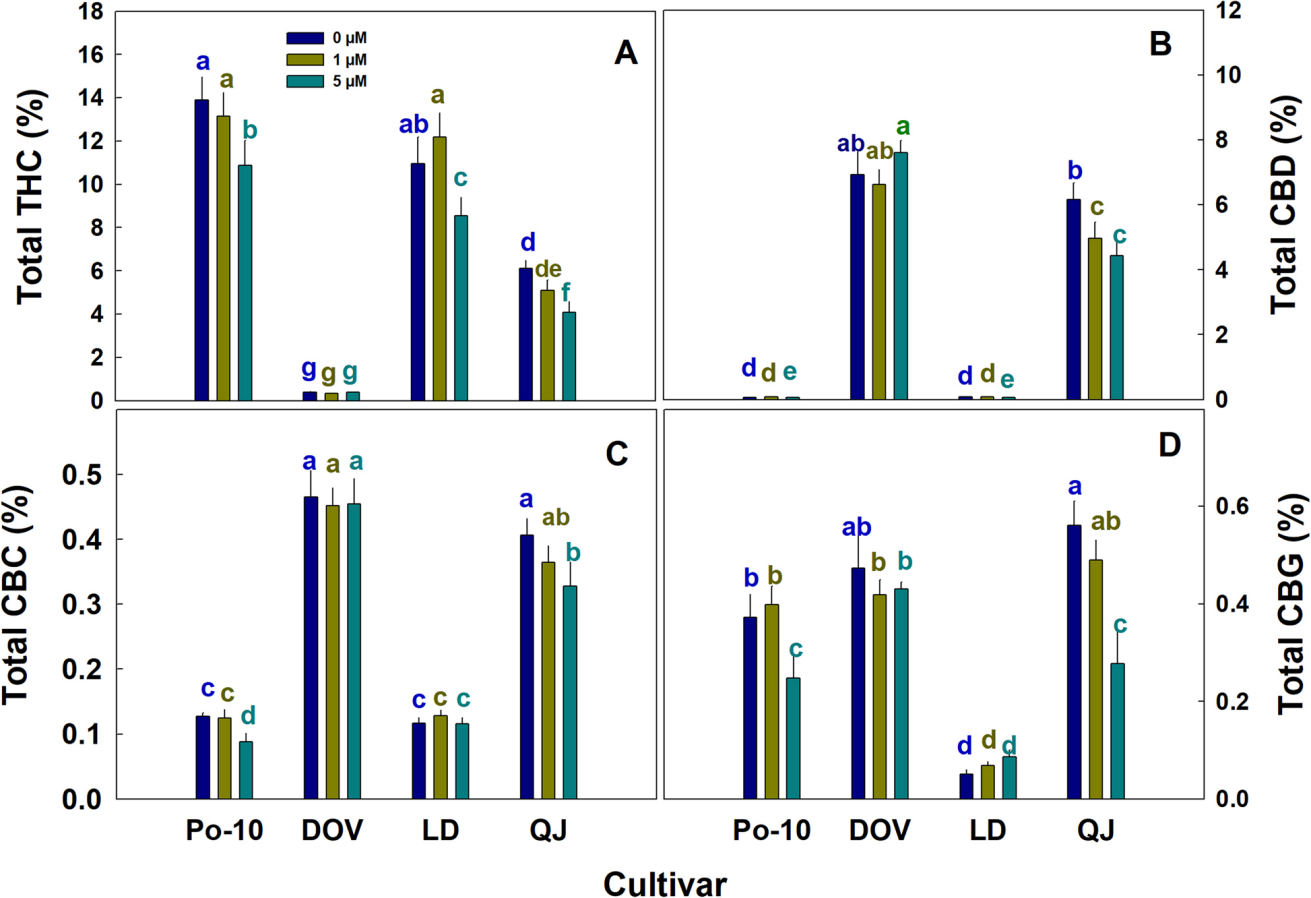
Effect of heavy metals on total cannabinoid concentrations (carboxylated+decarboxylated form of the cannabinoid) in inflorescences of four medical cannabis cultivars: QJ, PO, LD, Dov. The plants were exposed to a mix of four heavy metals (Pb, Co, Cd, Ni) at three concentrations (0, 1, 5µM). Total THC (**A**), total CBD (**B**), total CBC (**C**), and total CBG (**D**). The presented results are average ± SE (*n* = 5), % W/W. Different letters above the means signify significant differences between means by Tukey HSD test at α = 0.05

Figures 4 and 5 present the effect of the heavy-metal treatments on the concentrations of the acidic (carboxylated) and natural (decarboxylated) forms of the 4 major cannabinoids (THCA, THC; CBDA, CBD; CBCA, CBC; CBGA, CBG) (Fig. 4), and of the total of the decarboxylated and non-carboxylated forms for each of these cannabinoids (Fig. 5). The analysis revealed a dose-dependent response to the imposed heavy metal treatments, with genotypic variability.

The cannabinoids are biosynthesized in the plants as acidic forms, which can be decarboxylated to the neutral active forms in the plant, during post-harvest processing, or prior to consumption. Under 5 µM heavy-metal exposure, the concentrations of all the acidic forms of the cannabinoids decreased in PO and QJ (Fig. 4A, C, E, G), whereas in LD, a decrease was observed only for THCA. This suggests that the threshold for heavy-metal effects on cannabinoid production lies between the low and high concentrations tested, and exposures to heavy-metal levels above this threshold lead to a decrease of cannabinoid concentration. In Dov, the concentrations of both the acidic and the natural cannabinoids forms were not affected by the heavy-metal treatments (Fig. 4), in PO, CBG and CBC were reduced under 5 µM, and in LD a decrease was observed only for CBD under 5 µM heavy metal exposure (Fig. 4D, F, H). In QJ, all four natural cannabinoids were reduced under the 1 µM heavy-metal treatment (Fig. 4B, D, F, H).

The results of the total cannabinoid contents (acidic+decarboxylated forms) demonstrate as well that not all cultivars were affected by the heavy-metal treatments, and the responses are cultivar-specific, and are dependent on the heavy metal level (Fig. 5). In PO, the concentrations of all major cannabinoids were reduced by the 5 µM heavy-metal treatment, and in QJ the same trend was observed except that total CBD decreased already under the 1 µM treatment (Fig. 5A-D). In LD, total CBG and CBC were not affected by the exposure to the heavy-metal treatments (Fig. 5C, D), whereas THC and CBD decreased under 5 µM (Fig. 5A, B), and in Dov, none of the major cannabinoids were affected by the heavy metal treatments.

### Accumulation of heavy metals in the plant organs

**Fig. 6:**
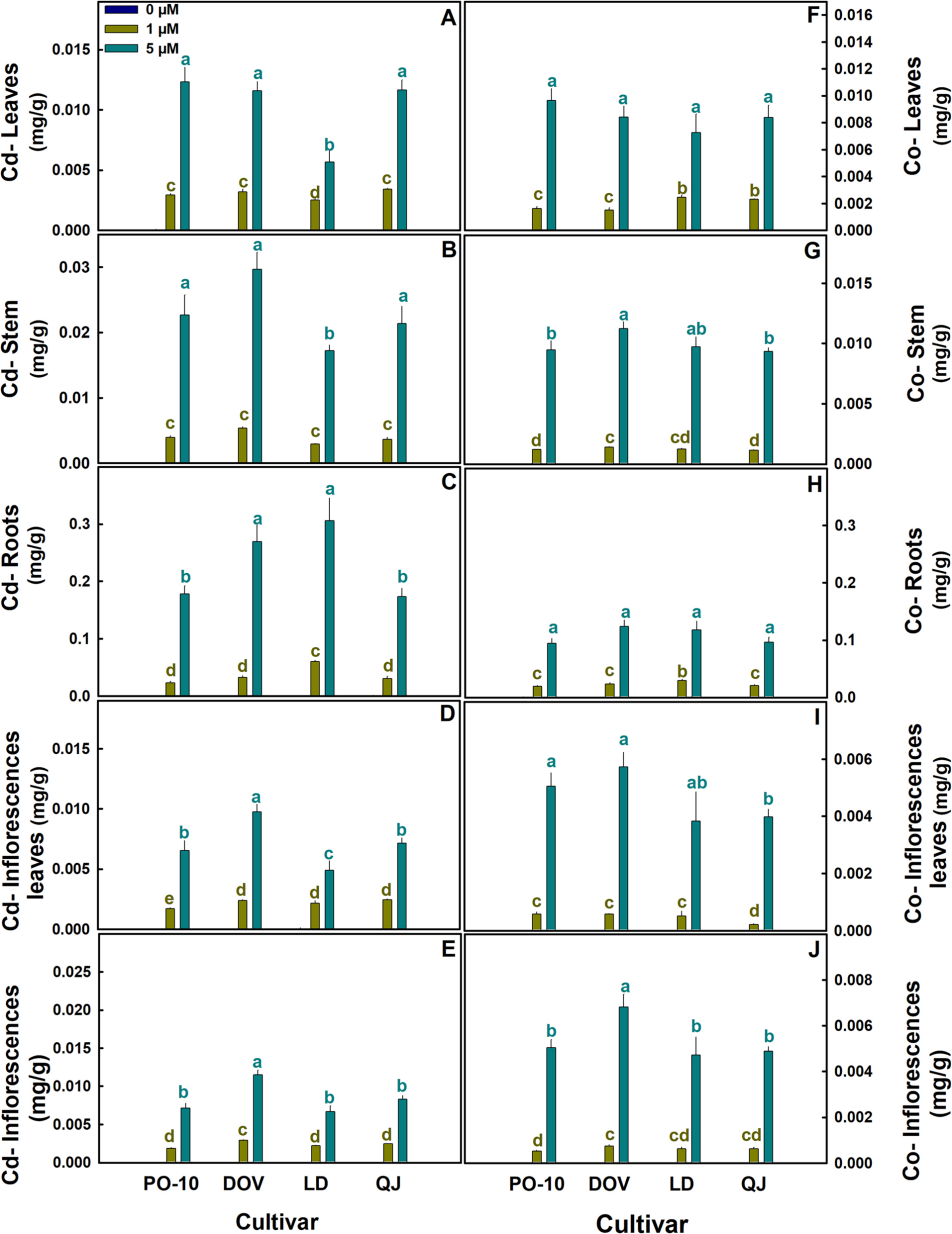
Effect of the heavy metal treatments on the accumulation of Cd and Co in the plant organs of four medical cannabis cultivars: QJ, PO, LD, Dov. The plants were exposed to a mix of four heavy metals (Pb, Co, Cd, Ni) at three concentrations each (0, 1, 5 µM). Cd-leaves (**A**), Cd-stems (**B**), Cd-roots (**C**), Cd-inflorescences leaves (**D**), Cd-inflorescences (**E**), Co-leaves (**F**), Co-stems (**G**), Co-roots (**H**), Co-inflorescences leaves (**I**), and Co-inflorescences (**J**). Presented results are average ± SE (*n* = 5). Where not presented, the heavy metal was not detected. Different letters above the means signify significant differences between means by Tukey HSD test at α = 0.05

**Fig. 7:**
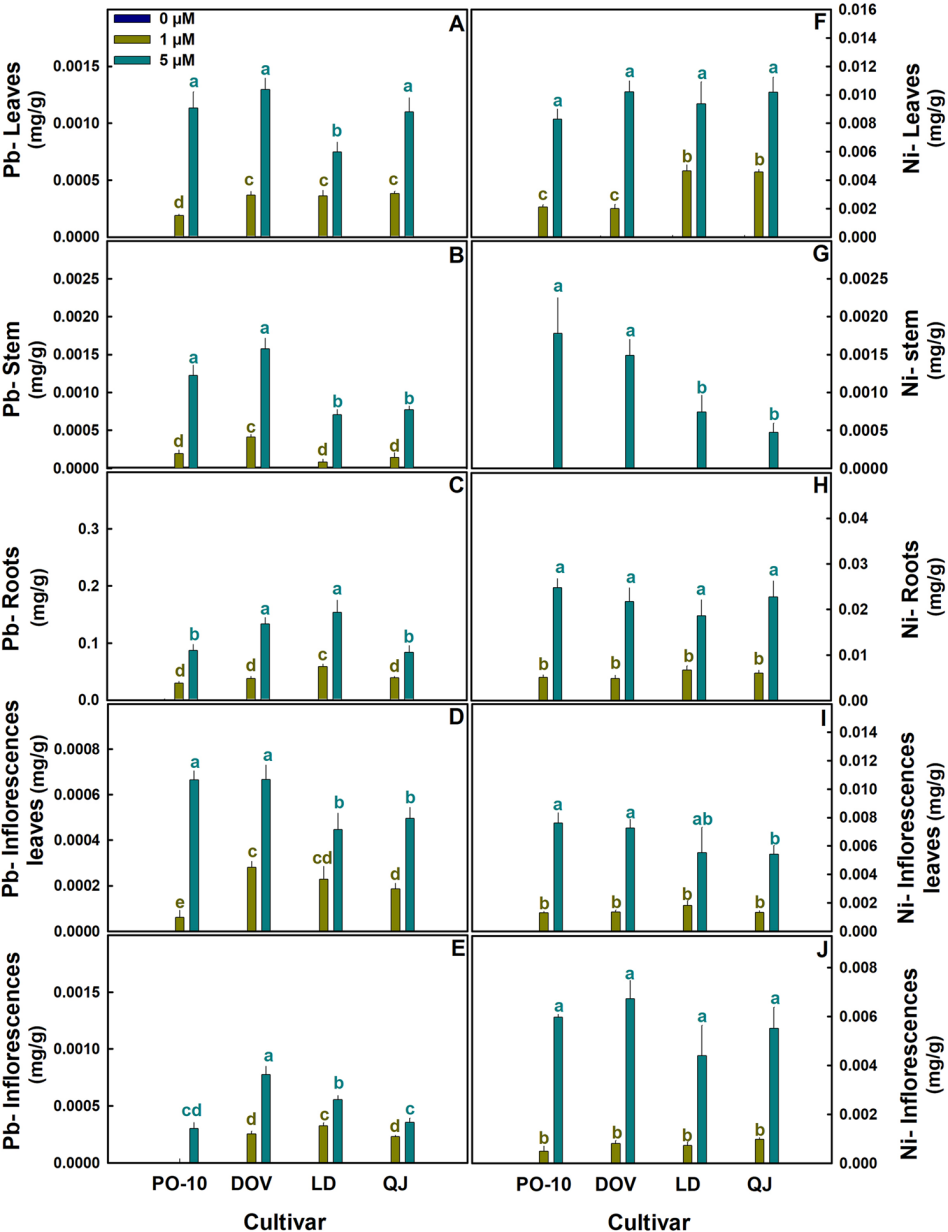
Effect of the heavy metal treatments on the accumulation of Pb and Ni in the plant organs of four medical cannabis cultivars: QJ, PO, LD, Dov. The plants were exposed to a mix of four heavy metals (Pb, Co, Cd, Ni) at three concentrations (0, 1, 5 µM). Pb-leaves (**A**), Pb-stems (**B**), Pb-roots (**C**), Pb-inflorescences leaves (**D**), Pb-inflorescences (**E**), Ni-leaves (**F**), Ni-stems (**G**), Ni-roots (**H**), Ni-inflorescences leaves (**I**), and Ni-inflorescences (**J**). Presented results are average ± SE (*n* = 5). Where not presented, the heavy metal was not detected. Different letters above the means signify significant differences between means by Tukey HSD test at α = 0.05

The accumulation of heavy metals in the plant organs was affected by the concentration supplied to the plants, with the responses differing between plant organs and cultivars. (Figures 6 and 7). The accumulation increased with increasing concentrations supplied to the plants; it differed between organs and was highest in the roots for all tested cultivars (Figs. 6C and H and 7C and H). Under the 5 µM treatment, the extent of accumulation in the plant organs was Roots> Stems> Leaves> Inflorescences leaves ≅ Inflorescences. E.g., it was highest at the vegetative organs (roots, stems, leaves) compared to the reproductive organs (inflorescences and inflorescence leaves), except for Ni, which accumulated to higher concentrations in the reproductive organs than in the stems, and furthermore, no Ni was detected in the stems under 1 µM heavy metals supply (Figs. 6 and 7).

**Fig. 8:**
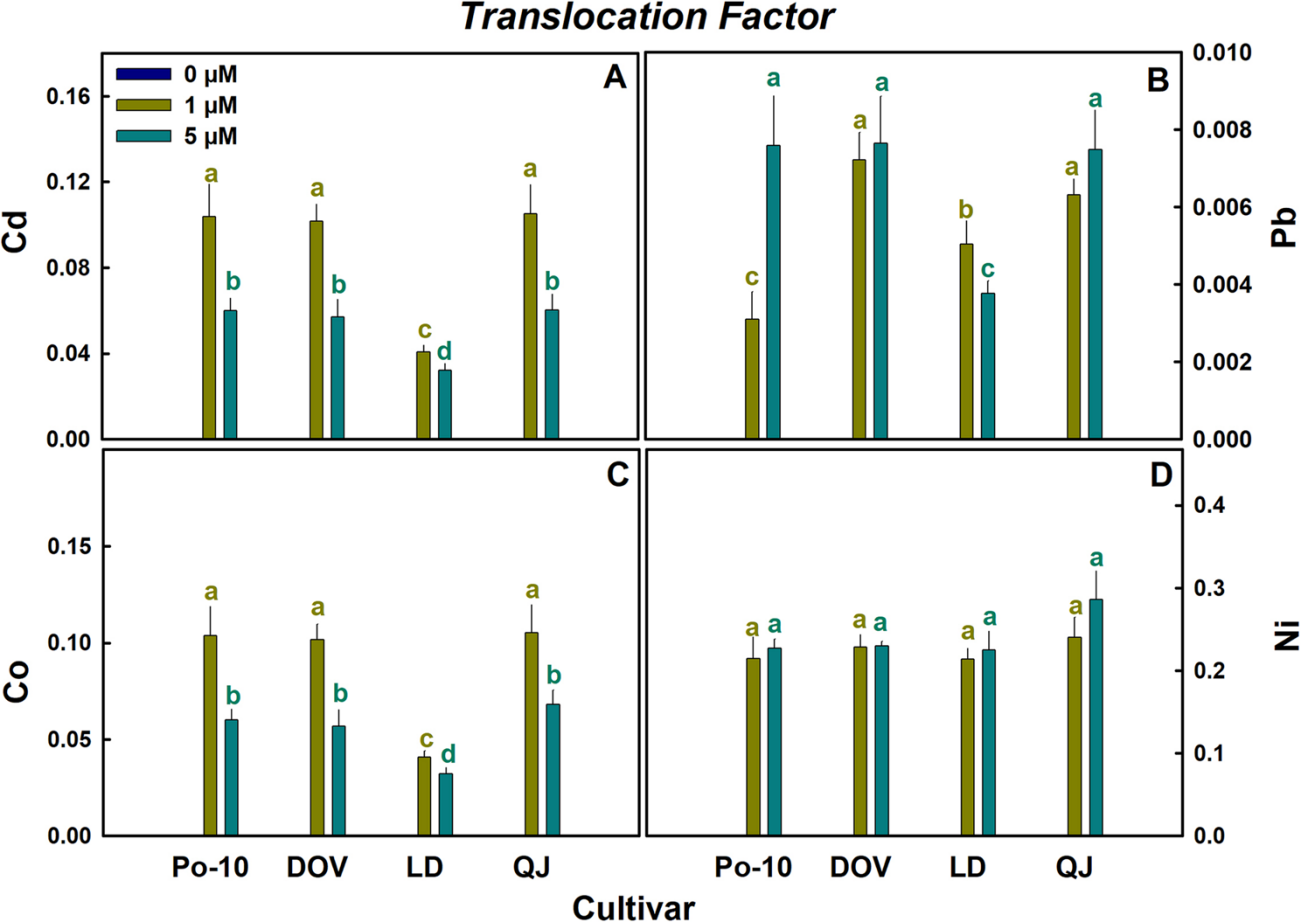
Effect of the heavy metal treatments on the translocation to the above-ground plant organs of the heavy metals Cd (**A**), Pb (**B**), Co (**C**), Ni (**D**), in four medical cannabis cultivars: QJ, PO, LD, Dov. The plants were exposed to a mix of four heavy metals (Pb, Co, Cd, Ni) at three concentrations each (0, 1, 5 µM). Presented results are average ± SE (*n* = 5). Where not presented, the heavy metal was not detected. Different letters above the means signify significant differences between means by Tukey HSD test at α = 0.05

The highest values for the translocation factor were obtained for Ni (Fig. 8D) and the lowest for Pb (Fig. 8B), indicating the highest and lowest root-to-shoot translocation, respectively. For both Co and Cd, the translocation factor was higher under the low heavy metal mix treatment (1µM) compared to the high concentration treatment (5µM), demonstrating that a larger proportion of the Co and Cd that were taken up by the plants remained in the root under the high concentration treatment (Fig. 8A). Different trends were observed for Pb and Ni. For Pb, in Dov and QJ the translocation factor was not significantly different between the two heavy metal treatments, but it was higher under the high treatment in PO, and higher under the low treatment in LD. For Ni, no significant differences in the translocation factors were observed between the high and low heavy metal treatments across all cultivars (Fig. 8D).

## Discussion

### Plant development and function

Heavy metals are well known to affect plant development and function via a range of mechanisms. While many heavy metals are toxic to plants, others are plant nutrients required for optimal plant functions and become toxic only at high concentrations. Some of the heavy metals which are not plant nutrients, are beneficial to plants, although they are not required elements.

Among the four heavy metals studied in the present project, Co and Ni are beneficial elements to plants at low concentrations, but under higher concentrations they are damaging to plants and can cause visible damage symptoms such as chlorosis, inhibit plant growth and development, inhibit biomass accumulation, and decrease contents of photosynthetic plant pigments (Shahzad et al. 2018; Hu et al. 2021). The other two heavy metals evaluated in the study, Pb and Cd, are non-essential elements for plants, that induce toxic effects even at low concentrations (Zulfiqar et al. 2019; El Rasafi et al. 2022).

Exposure of the cannabis plants to the heavy metal cocktail induced development of chlorosis symptoms under the high concentration treatment (5 µM) (Fig. 1), corresponding to the response of numerous plants to heavy metal exposure (Khan and Khan 2010; Zayneb et al. 2015). The heavy-metal treatments did not affect morphological development and biomass accumulation in any of the cultivars tested (Fig. 2), demonstrating that the highest concentration of the heavy metal imposed on the plants (5µM) is below the toxicity level for drug-type cannabis.

The heavy metal treatments did not affect gas exchange activity in three of the four cultivars tested; in the QJ cultivar, they reduced net photosynthesis rate, stomatal conductance, and transpiration rate (Fig. 3A-C). The decrease in gas exchange activity may have resulted from damaging effects of Cd, as Cd is known to hamper gas exchange activity and water relations by reducing CO_2_ intake and disrupting stomatal regulation thus reducing net photosynthesis rate (El Rasafi et al. 2022). Since we have studied the effects of a mixture of several heavy metals, the effects on the leaf gas-exchange activity might result from their combined effects. Interactive effects of heavy metals are indeed known to affect plant response to heavy metal toxicity. For example, simultaneous application of Pb and Cd is known to induce oxidative stress as well as the activity of antioxidant defense enzymes (Ahmad et al. 2012), and a simultaneous exposure to Co and Cd can decrease ethylene activity whose synthesis is induced under Cd exposure as a defense mechanism against the stress (Chmielowska-Bąk et al. 2014). The reduction in photosynthesis can be related also to the exposure to Ni, as exposure to excessive Ni concentrations inhibits key enzymes of the Calvin cycle (Shahzad et al. 2018). Ni and other heavy metals including Pb were reported to negatively affect gas exchange activity, photosynthesis and concentration of photosynthetic pigments under exposure to a concentration above a certain level (Bazzaz et al. 1974; Fu and Wang 2015; Aguilar et al. 2023), demonstrating again a dose response. The slight improvement in gas exchange parameters under Ni exposure can be explained by the importance of Ni for the function of the enzyme hydrogenase (Shahzad et al. 2018), which is crucial for the electron transport chain.

Contents of photosynthetic pigments in the plants were reduced as well by the heavy metal treatments in two of the tested cannabis cultivars (Fig. 3E-G). This is in accord with the negative impact of heavy metals on the accumulation of photosynthetic pigments in different plants (Petrović et al. 2024; Tang et al. 2015). Heavy-metal induced reduction in pigments’ accumulation can be related to a range of mechanisms and processes, including Ni-stress induced deficiency of Mg and Fe and the resulting inhibition of chlorophyll biosynthesis (Shahzad et al. 2018); damaging effects of Co on chloroplast membrane structure and thus inhibition of PS2 activity (Palit et al. 1994); Pb inhibition of chlorophyll biosynthesis by inhibition of the activity of key enzymes involved in the production of the pigments (Collin et al. 2022); and Cd induced production of chlorophyllase, which degrade the chlorophyll molecule (El Rasafi et al. 2022). The mechanisms resulting in reduced pigment content in cannabis under exposure to the studied heavy metals, and the basis for the variable tolerance between cultivars, are not known and require further study.

Our results, which identified damaging effects of the heavy metals on gas exchange activity in only one out of four varieties, and on photosynthetic pigments in only two of the cultivars (Fig. 3), reveal differential genotypic sensitivity to heavy metals in ‘drug-type’ cannabis.

### Cannabinoid profiles

The heavy metal treatments affected cannabinoid concentrations in the inflorescences (Figs. 4 and 5). The effect varied between cultivars, demonstrating genotypic variability also in the sensitivity of cannabinoid metabolism to the imposed heavy metals. Specifically, the total contents of all major cannabinoids (i.e., the normalized sums of the acidic and decarboxylated forms) were not affected in Dov, decreased in QJ and Po, and in LD decrease only for THC and CBD (Fig. 5).

Inhibitory effects of the heavy metals on the production of secondary metabolites were reported for several plants and secondary metabolites. Specifically, biosynthesis of flavonoids, phenols and saponins decreased in *Gynura procumbens* by exposure to Cd and Cu (Ibrahim et al. 2017), and exposure to Ni reduced hypericin and pseudohypericin production in *Hypericum perforatum* L. (Murch et al. 2003). But the inhibitory effects are not inclusive- exposure of *Ocimum basilicum* L. plants to Cd, Pb and Cr increased production of methyl-chavicol and decreased linalool content; whereas linalool and methyl-chavicol decreased, and methyl-eugenol increased by exposure of these plants to Ni (Prasad et al. 2011). Increased production of secondary metabolites under exposure to heavy metals was reported also for carvone in *Mentha crispa* L. under Pb exposure (Sá et al. 2015), and diosgenin in *Trigonella foenum*-*graecum* under exposure to Cd and Co (De and De 2011). In ‘fiber-type’ Hemp, CBD and THC contents decreased under exposure to Cd (Marabesi et al. 2023b).

Our results revealed a genotypic dependency in the response of the major cannabinoids to heavy metals; and differences between cannabinoids in the concentration threshold for heavy metal effect on production of cannabinoids, as concentrations of some of the cannabinoids were affected only under the high heavy metal concentration (5 µM), but not under the low concentration (1 µM) (Figs. 4 and 5). Heavy metal stress is known to stimulate ROS production, and oxidative stress was demonstrated to affect secondary metabolites production (Berni et al. 2019). There are indications in our results that in cannabis as well, the heavy-metal induced alterations in cannabinoid metabolism are related to oxidative stress: QJ was the only cultivar that suffered an increase in membrane leakage under the heavy metal exposure (Fig. 3H), which is considered a sign of membrane lipid peroxidation due to oxidative damage, and this cultivar suffered the most negative effects on cannabinoid production. The extent of reduction in total cannabinoid contents and the acidic forms of the cannabinoids was accordingly generally higher for this cultivar compared to the other three cultivars tested (Figs. 4 and 5).

### Contamination of the cannabis yield and plant organs by the heavy metals

The extent of heavy-metal accumulation in the plants reflects the concentrations available for plant uptake, as the accumulation increased with increasing metal concentrations in the root solution (Figs. 6 and 7). The highest accumulation of the heavy metals was found in the root (Figs. 6 and 7), demonstrating sequestration- an avoidance strategy to cope with toxic metals by excluding them from sensitive organs and cellular compartments such as leaves and cytoplasm, into less sensitive organs or compartments such as roots and vacuoles, or by efflux from the cell.

Cd accumulation in the plant was the highest among the tested heavy metals (Figs. 6 and 7), likely due to low ion competition at the uptake sites of the root, and the ability of Cd to cross the root cell membrane passively, via non-specific membrane transport proteins (Yan et al. 2017; Sterckeman and Thomine 2020; El Rasafi et al. 2022). Plants have various strategies to minimize Cd absorption and limit its’ transfer to sensitive apical organs (El Rasafi et al. 2022). The reduction of the root: shoot translocation factor with the increase in heavy-metal concentration, coupled with the high level of Cd accumulation in the roots, demonstrates an avoidance strategy of Cd compartmentation in the root in all the cultivars tested.

The root-to-shoot translocation factor of Ni was the highest among the heavy metals (Fig. 8). Ni is known to exhibit efficient root-to-shoot translocation in plants due to its association with various root-to-shoot metal transporters that transport Ni as well (Fadzil et al. 2024); and its affinity to the imidazole ring in histidine and nicotianamine, a chelation that supports its root-to-shoot translocation (Amari et al. 2017).

Lead (Pb) had the lowest root to shoot translocation factor among the tested heavy metals (Fig. 8). This trend of restricted Pb translocation to the aerial parts of the plant is likely due to its compartmentation in vacuoles facilitated by numerous transporters and proteins (Lee et al. 2005; Zhu et al. 2013), and binding to ion-exchange sites at the cell wall, as Pb accumulation can lead to expansion of the vacuoles and cell wall widening thus enhancing the binding potential (Rucińska-Sobkowiak et al. 2013).

The distribution pattern of Co in the plant was similar to that of the other heavy metals, with a translocation factor (TF) similar to Cd, i.e., lower than for Ni and higher than for Pb (Figs. 6, 7 and 8). Similar to Cd, the Co-TF was lower at the high (5 µM) than the low (1 µM) heavy metal treatment, and this is consistent with the higher concentrations of Co and Cd in the plant roots under the high compared to the low heavy metal treatment (Figs. 6 and 8), suggesting a restriction of xylem loading. These findings suggest that in cannabis, only a small portion of the Co taken up by the cannabis roots is loaded into the xylem and transported to the shoot, for accumulation in other plant organs. A similar trend for root accumulation of Co was identified in wheat (Page and Feller, 2005). Not much information is available about Co in the literature; it has a few beneficial roles in some plants (Hu et al. 2021). And due to the potential for contamination of the crop yield, with resulting hazards to consumer health, it is important to further explore this issue.

The heavy metals supplied to the plants (Cd, Co, Ni, Pb) translocated to the reproductive organs- inflorescences and inflorescence leaves (Figs. 6 and 7). It is known that trichomes have a role in accumulation and detoxification of heavy metals (Li et al. 2023). The trichomes’ basal cells are linked through plasmodesmata to epidermal and mesophyll cells, which are connected to the vascular tissues (Čiamporová et al. 2021); and transporters and chelating ligands are present in the trichomes (Gao et al. 2021; Li et al. 2021) facilitating heavy metal transport from the vascular system to the trichomes and localized accumulation. Thus, plant trichomes can translocate heavy metals into and out of cells. This mechanism takes part in metal homeostasis in the cells (Li et al. 2021; Ricachenevsky et al. 2021), defense against herbivores (Stolpe et al. 2017), and detoxification- as heavy metal may be excluded from the trichomes by forming crystals that consist of high concentrations of the heavy metals thereby increasing tolerance to toxic amounts of the heavy metals (Choi et al. 2001). In cannabis, the commercial product consumed by the patients are the inflorescences, which contain the highest density of trichomes in the plant. It is therefore very important to understand the fate and homeostasis dynamics of heavy metals in these reproductive organs, to facilitate the development of cultivation regimes optimized for safe product. Not much information is available on this subject. Since tobacco plants can excrete heavy metals through their trichomes (Sarret et al. 2006), it is likely that so do cannabis plants. Seeds of hemp-cannabis are indeed known to accumulate heavy metals, including Cd (Mihoc et al. 2012) and Cr (Eboh and Thomas 2005), which implied that transport, accumulation, and sequestration of heavy metals in cannabis involves its reproductive organs.

Among the four cultivars tested, Dov accumulated the highest concentrations of heavy metals, or was among the highest-accumulating varieties (Figs. 6 and 7). Industrial ‘fiber-type’ cannabis (hemp) is well known for its high ability for root uptake and plant accumulation of heavy metals, and it is thus explored for its phytoremediation potential (Placido and Lee 2022). Interestingly, in our study, the cultivar that presented the highest accumulation of the heavy metals was Dov, which has a typical hemp-type CBD-rich cannabinoids profile. For practical phytoremediation use, it is best to utilize plants that produce high biomass, and are thus capable of removing a large quantity of the toxic metals with the developed biomass. The Dov cultivar accumulated the highest concentrations of the heavy metals, but its biomass production was lowest (Fig. 2D-H); therefore practical use of its high phytoremediation potential will require breeding for higher vigor.

The World Health Organization (WHO) published maximum limits of toxic metals concentrations in medicinal plants and their products (WHO, 2007). According to these guidelines, the limits of Pb, Cd, Ni in herbal medicines and products are 10, 0.3, and 1.5 µg/g, respectively. When comparing our results to the maximum limits published by the WHO, the concentrations of Pb accumulated in the inflorescences were below the limits under the 5µM treatment (0.301, 0.775, 0.354, 0.353 µg/g for PO, Dov, LD and QJ, respectively), but Cd and Ni exceeded the maximum limits in all four cultivars under the same treatment (7.13, 11.5, 6.67, 8.3 µg/g of Cd and 5.96, 6.71, 4.39, 5.51 µg/g of Ni for PO, Dov, LD and QJ, respectively). These findings, which present accumulation of heavy metals in the drug-type cannabis inflorescences to concentrations higher than permitted by the WHO for medicinal plants consumption, raise health concerns and point to the need for further study of heavy metal dynamics in medical cannabis cultivation.

## Conclusions

The results confirm the effects of heavy metals on cannabinoid production, with a heavy-metal concentration threshold, and genotypic variability, thus confirming the hypotheses. The effects of heavy metals on the tested cultivars ranged from a considerable negative impact on cannabinoid production and plant function, to no effect. Our results also revealed variability among the major cannabinoids in their responses to heavy metals in some of the cultivars tested, as well as variation in the concentration threshold for heavy metal effects on cannabinoids production among the cannabinoids.

The accumulation of heavy metals varied between cultivars and organs. Interestingly, the CBD-rich type cultivar Dov, was the only cultivar that suffered no negative effects on cannabinoid production, and furthermore, was among the cultivars that accumulated the highest concentrations of heavy metals, including in the inflorescences, similar to fiber-type cannabis (hemp). The inflorescence concentration of two of the four heavy metals tested exceeded the WHO-permitted threshold for medical plant consumption. These alarming results should be considered in developing world guidelines for cannabis cultivation, to prevent exposure of consumers to toxic levels of these metals.

## Data Availability

All data is available in the manuscript.
